# Pilot Testing of a Sampling Methodology for Assessing Seed Attachment Propensity and Transport Rate in a Soil Matrix Carried on Boot Soles and Bike Tires

**DOI:** 10.1007/s00267-016-0773-4

**Published:** 2016-10-17

**Authors:** Nigel Hardiman, Kristina Charlotte Dietz, Ian Bride, Louis Passfield

**Affiliations:** 1 School of Anthropology and Conservation, University of Kent, Canterbury, Kent CT2 7NZ UK; 2 Lincoln International Business School, University of Lincoln, Brayford Pool, Lincoln LN6 7TS UK; 3 School of Sport and Exercise Science, University of Kent, Canterbury, Kent CT2 7NZ UK

**Keywords:** Weeds, Seed attachment, Human-mediated dispersal, Tourism impacts

## Abstract

Land managers of natural areas are under pressure to balance demands for increased recreation access with protection of the natural resource. Unintended dispersal of seeds by visitors to natural areas has high potential for weedy plant invasions, with initial seed attachment an important step in the dispersal process. Although walking and mountain biking are popular nature-based recreation activities, there are few studies quantifying propensity for seed attachment and transport rate on boot soles and none for bike tires. Attachment and transport rate can potentially be affected by a wide range of factors for which field testing can be time-consuming and expensive. We pilot tested a sampling methodology for measuring seed attachment and transport rate in a soil matrix carried on boot soles and bike tires traversing a known quantity and density of a seed analog (beads) over different distances and soil conditions. We found % attachment rate on boot soles was much lower overall than previously reported, but that boot soles had a higher propensity for seed attachment than bike tires in almost all conditions. We believe our methodology offers a cost-effective option for researchers seeking to manipulate and test effects of different influencing factors on these two dispersal vectors.

## Introduction

Invasive alien species of plants (weeds), together with animals, fungi and microbes are widely recognised as posing a major threat to global biodiversity, second only to habitat destruction in their impact (Randall 1996; Vilà et al. 2011; Wittenberg and Cock 2001; World Conservation Union [IUCN] 2000). Weeds have been shown to cause billions of dollars of annual economic loss in agriculture and forestry (Pimentel et al. 2001; Pimentel 2002; Williams et al. 2010). They have also been shown to alter ecological processes, degrade ecosystem services and disrupt ecological integrity (DiTomaso 2000; Mack and D’Antiono 1998; Pejchar and Mooney 2009; Pimentel 2002; Williams et al. 2010). Dispersal of weeds can occur via a variety of diaspores, including as adult individuals, ramets, bulbs or seeds, and can be mediated both by natural vectors, e.g., wind, rain, flowing water, animals, by humans or a combination of these (Nathan 2006; Ridley 1930; Wichmann et al. 2009). Studies have shown that dispersal of even small numbers of seeds, especially over large distances, can cause disproportionally large changes in ecological patterns (Cain et al. 2000; Higgins et al. 2003; Nathan 2006).

One human activity with high potential for unintentional dispersal of weed seeds is tourism (including recreation). People today, especially in economically developed countries, have increasing time for leisure (Molitor 2000) and international tourism has demonstrated rapid and almost continual growth in recent decades, with over 1 billion international tourists recorded in 2012 (UNWTO 2013). Risk of human-mediated dispersal of seeds by recreation may be especially important in protected natural areas, where it may be one of only a few human activities allowed (Newsome et al. 2002; Worboys et al. 2005) and where introduced seeds may develop into invasive environmental weeds. Research has shown an association between weed presence and tourism infrastructure in natural areas, especially adjoining roads and tracks (Pickering et al. 2007; Potito and Beatty 2005; Spellerberg 1998) and increasing weed diversity with increasing tourist visitation (Usher 1988).

A small but growing number of studies have shown capacity for unintentional human-mediated dispersal of seeds by tourists, either attaching directly to hikers’ clothing or equipment, embedded in soil picked up by vehicles, or animal dung/feed (for comprehensive reviews see Pickering and Mount 2010; Ansong and Pickering 2013 and 2014). The number of seeds dispersed by such vectors can be large (e.g., ≈1300 on a walker’s socks after only a 5 min hike through roadside vegetation: Mount and Pickering 2009) and of high species richness (e.g., >750 species collected from various tourism-related vectors: Pickering and Mount 2010), of which a high proportion have typically been subsequently identified as national or international invasive species (Mount and Pickering 2009).

Despite such demonstrated potential, controlled experiments to quantify propensity for seed attachment and/or dispersal by people while hiking, either attaching directly to clothing or embedded in a soil matrix carried on boot soles, are scarce. We found only two studies that experimentally tested direct seed attachment rates on human skin/clothing (boots, socks, laces and trousers: Falinski 1972; boots, socks, laces, trousers and bare legs: Mount and Pickering 2009) and only a single study of seed attachment in a soil matrix carried on boot soles: Wichmann et al. 2009). We also found only four studies that experimentally tested dispersal of seeds attaching directly to clothing (trousers and shirts: Bullock and Primack 1977; boots, socks, outer clothing and personal luggage: Lee and Chown 2009; trousers and socks: Ansong et al. 2015; Pickering et al. 2011) and a single study of seed dispersal via a soil matrix on boot soles (Wichmann et al. 2009). Even within the few aforementioned experimental studies on seed attachment on boots, relatively few factors affecting attachment rates appear to have been tested, i.e. distance walked (Falinski 1972), trousered vs. bare leg (Mount and Pickering 2009) and seed species, individual walkers and boot types (Wichmann et al. 2009). Research on the effects of other potentially important factors, for example seed size, mass and morphology, soil type and condition (e.g., wet vs. dry), appears to be scarce.

Alongside hiking, another recreation activity with high potential for weed seed introduction and/or dispersal is off-road cycling (‘biking’) (Pickering et al. 2010). Biking is increasingly popular globally in backcountry/wilderness protected areas such as national parks (Burgin and Hardiman 2012; Hardiman and Burgin 2013) and in open access peri-urban natural areas (Chiu and Kriwoken 2003) and its growth has led to increasing user group pressure for greater access to natural areas. Although a small number of experimental studies have attempted to measure biking’s absolute and relative potential (e.g. vs. hiking) for direct environmental degradation of such factors as increased soil exposure, decreased vegetation cover and/or species richness [e.g., Newsome and Davies (2009), Pickering et al. (2011), Thurston and Reader (2001)], no published studies to date have experimentally tested seed attachment or dispersal propensity on mountain bike tires, either in absolute terms or relative to boot soles.

The propensity for attachment and dispersal of seeds in a soil matrix on boot soles or bike tires is likely to differ for many reasons. Some key variables include: (i) available surface area of soles vs. tires [tires larger than boots (Thurston and Reader 2001)]; (ii) ground contact pattern (boots: discrete steps and equal distance covered by each boot; tires: continuous contact and different ground contact distance covered by front and rear tires); (iii) ground contact pressure [biker higher than walker (Thurston and Reader 2001)]; (iv) different tread patterns and depth of soles/tires; (v) distance covered (bike riders typically travel faster and further than walkers for a given time/effort); (vi) soil type and; (vii) soil condition (e.g. moisture content). The number and density of seeds available for attachment, along with differences in their size, morphology, weight and surface adhesion qualities, also potentially affect their attachment and/or dispersal rate. Field testing of such multiple variables is typically time-consuming and expensive. Researchers therefore need a sampling methodology that allows control of such variables while still representing ‘real world’ behaviour. This study sought to fill an existing knowledge gap by testing a potential sampling methodology for experimentally testing the absolute and relative propensity for seed attachment and transport in a soil matrix (a) on boot soles and bike tires (b) in wet or dry soil (c) over different distances travelled.

## Methods

### Procedure

We constructed a circular, prefabricated track measuring 0.75 m wide with 50 mm sidewalls and external radius of 2.75 m and internal radius 2.0 m, giving a track centre line circumference of 14.92 m and surface area of 11.18 m^2^.The track was designed to simulate the width of a typical outdoor trail and allow for a normal walking and cycling movement. Testing of different track widths and circumferences showed that this was the smallest size in which a typical bike could be ridden in a ‘normal’ fashion (i.e. without the riders’ feet or hands touching the ground or a wall for balance support).

In real world conditions, the number and/or density of seeds available for attachment and dispersal is likely to be highly variable and affected by many external factors; definition of what is a ‘realistic’ and ‘biologically-relevant’ number and/or density is therefore situation-specific. To provide a benchmark, however, we designed our seed/soil density to be comparable to that used in the experiment by Wichmann et al. (2009). The aims and sampling methodologies of the two experiments were very different, however. In Wichmann et al.’s (2009) study, the researchers’ primary focus was on measuring seed dispersal rate carried in a soil matrix in boot soles over distance, and their sampling protocol aimed to maximise initial seed attachment. They used 500 g (volume unspecified, probably ~0.5 l) of a ‘sandy silty loam’ soil, oven dried at 30 °C, spread evenly in a tray (400 mm × 250 mm; soil depth unspecified), wetted with 50 ml of water using a plant mister and stirred (moisture level unspecified). A walker then placed both shoe-clad feet in the tray and took 20 steps on the spot to pick up soil. The walker then stepped into a second tray (unspecified; assumed to be of same dimensions as Tray 1) containing 100 evenly spread seeds, either *Brassica oleracea* [wild cabbage] or *Brassica nigra* [black mustard], again taking 20 steps on the spot. Assuming Tray 1 was filled to a soil depth of 20 mm and Tray 1 and Tray 2 were of equivalent dimensions, this would suggest a soil area of 100,000 mm^2^ and density of seeds 100/100,000 mm^2^ = 0.001 seeds/mm^2^, although the actual density of seeds exposed to the boot soles was probably much higher than this: ‘probably artificially high’ (Wichmann et al. 2009, p. 525, 530). The number of seeds attaching was calculated by subtracting the number left in the tray from 100, yielding the pickup rate (Wichmann et al. 2009, p. 524).

We used:

1. 240 l of soil spread evenly on the sampling track to an approximate depth of 20 mm (0.02 m depth × 11.18 m^2^ area = 0.2236 m^3^). We used a commercially-obtained loam-based soil (“J. Arthur Bower’s Topsoil” TM: William Sinclair Horticulture Limited 2008).
2. 11,180 ‘seeds’ (11.18 m^2^ area × 0.001 seeds/mm^2^ = 11,180), i.e. 50 ‘seeds’/l of soil [vs. at least 200 seeds/l of soil in Wichmann et al. (2009)]. Wichmann et al. (2009) used a *Brassica*-species seed, artificially coloured to aid on-ground identification. As artificially colouring the much larger quantity of seeds we used was impractical, we used synthetic ‘seed beads’ (‘Size 11 Japanese Toho’ TM: Product code 11R43F; Beads Direct 2013), purchased in a bright blue colour. The beads were roughly spherical in shape and sampling measurements showed a mean maximum diameter 2.1 mm (SE = 0.07 mm) and mean minimum diameter 1.6 mm (SE = 0.09 mm), making them comparable in size and shape to the *Brassica* spp. employed by Wichmann et al. (2009). The beads were sprinkled evenly over the soil surface and mixed in by light raking before each sampling replicate.

The sampling track was set up indoors on the University of Kent’s Canterbury campus and sampling was undertaken on the 4th, 6th and 7th September, 2013.

### Design

The experiment was a 2 × 2 × 2 factorial design with factors Vector (“boot” vs. “bike”), Soil Condition (“moist” vs. “wet”) and Traversal Distance (“short” vs. “long”). For operational reasons (e.g., “wet” and “moist” could not be randomised), testing followed a systematic sampling order: boot, moist, short; boot, moist, long; bike, moist, short; bike, moist, long; boot, wet, short; boot, wet, long; bike, wet, short; bike, wet, long. The complete sequence was replicated seven times.

The Vector “Boot” comprised one pair of newly-purchased general purpose wellington boots (“Traditional Green PVC Wellington Boot”, British size 8, heel/sole tread depth 10/5 mm; Briers 2011). “Bike” was a “hybrid” road/off road bicycle with side-pull caliper brakes and new tires (Claud Butler “Urban 2000’ 18” frame with Meghna “Explorer” 700 × 38 mm tires, with a tread depth 2 mm).

Soil condition (MEA 2013) was measured at the beginning, middle and end of each testing day, using a Lutron soil moisture meter PMS-714 (Lutron Undated). “Moist” soil ranged between 18.7–21.6 % during testing. After completion of moist testing, water was mist sprayed incrementally and evenly onto the soil from a handheld garden sprayer and “wet” soil was >50 % (moisture metre maximum reading) throughout testing.

The Traversal Distance “short” test comprised one complete circuit of the track (≈15 m) and a “long” test comprised 10 circuits (≈150 m). Walking circuits were standardised to 25 discrete paces/circuit (both feet combined). The same team member completed all walks and rides in an anticlockwise direction.

On completion of each designated walk/ride distance, the walker stepped/bike was lifted carefully into a sorting tray measuring 2300 × 500 × 50 mm with a bright white base. Then during a timed 10 min period all the soil and beads adhering to boots/tires were carefully brushed off. The beads were found (facilitated by their bright blue colour) and counted by team members using LED head torches and magnifying glasses. After counting, beads were cleaned and, together with the soil from the sorting tray, sprinkled evenly back around the track and the soil was raked over before commencing the next test.

### Analyses

As the outcome variable, the number of beads attaching, is a non-negative count, data were analysed using (i) one-way ANOVA for testing bead attachment rate between left vs. right boot soles and front vs. rear bike tires; and (ii) count models (Hilbe 2011; Ridout Demétrio and Hinde 1998). For testing main and interaction effects of the three factors: Vector (Boots; Tires), Soil Condition (Moist; Wet) and Traversal Distance (Short; Long), replicate number was also included in the analysis as a blocking factor, but was not significant. Poisson and negative binomial count models were considered. For several of the eight treatment combinations, variation between replicate counts was much greater than would be expected if counts followed a Poisson distribution. Due to this over-dispersion, a negative binomial model was used for analyses of the three factors. Analyses were conducted in R, version 3.1.1 (R Core team 2014). Results were accepted as significant at or below the 5 % probability level.

## Results

Beads were only recorded attaching to boots and tires along with soil; no “bead-only” attachment was recorded under any sampling parameter combination. We observed that boots predominantly tended to pick up soil and beads in the heel treads, with soil tightly compacted and requiring beads to be physically extracted by the researchers, with very few beads (estimated <5 %) attaching to the remainder of the soles. One-way ANOVA testing revealed no significant difference in bead attachment quantity or % rate between left and right boots for all parameter combinations (*F*_1,54_ = 1.49, *P* = 0.23). In contrast, bike tires showed a significant difference (*F*_1,54_ = 15.30, *P* < 0.0003) in bead attachment quantity and % attachment rate between front and rear tires, with attachment on the front tyre at least an order of magnitude higher than the rear for all sampling parameter combinations except “short traversal, moist soil” (zero bead attachment recorded on both tires for all replicates, see Tables 1 and 2).

**Table 1:** Summary of results showing absolute and comparative propensity for bead attachment (observed data) on boot soles and bike tires over seven replicated tests

	Moist				Wet			
	Total # beads attaching	% of total beads attaching all tests	M # (SE) of total beads attaching	Mean % attachment of beads available (SE)	Total # beads attaching	% of Total beads attaching all tests	M # (SE) of total beads attaching	Mean % attachment of beads available (SE)
Boot short left	19				48			
Boot Short right	35				89			
Boot short total	54	6.7	7.7 (1.82)	0.07 (0.02)	137	16.9	19.6 (3.78)	0.18 (0.03)
Boot long left	39				88			
Boot long right	37				85			
Boot long total	76	9.4	10.9 (1.37)	0.10 (0.01)	173	13.2	24.7 (3.25)	0.22 (0.03)
Bike short front	0				100			
Bike short rear	0				7			
Bike short total	0	0.00	0.0 (0.00)	0.00 (0.00)	107	21.4	15.3 (5.13)	0.14 (0.05)
Bike long front	19				230			
Bike long rear	2				12			
Bike long total	21	2.6	2.9 (0.83)	0.03 (0.01)	242	29.9	34.6 (4.42)	0.31 (0.04)

Note: (i) Total number of beads attaching over all tests = 810; (ii) Total number of beads available for attaching per test = 11,180

**Table 2:** Summary of raw data showing actual number of beads attaching on boot soles and bike tires by treatment and replicate

Boot soles							
Left moist short	Right moist short	Left moist long	Right moist long	Left wet short	Right wet short	Left wet long	Right wet long
0	0	3	5	3	4	7	8
2	2	12	6	3	14	7	14
4	3	6	3	18	11	20	22
3	5	4	4	0	11	16	10
2	7	4	9	1	18	12	8
6	9	6	5	3	15	14	8
2	9	4	5	20	16	12	15

Bike tires							
Front moist short	Rear moist short	Front moist long	Rear moist long	Front wet short	Rear wet short	Front wet long	Rear wet long
0	0	1	0	10	5	39	0
0	0	6	0	16	0	42	0
0	0	1	0	17	1	34	1
0	0	4	0	43	0	20	3
0	0	2	0	6	0	28	6
0	0	3	2	7	0	17	0
0	0	2	0	1	1	50	2

Total number of beads available for attaching per test =11,180

The negative binomial model provided adequate fit for the data; that is predicted seed-counts did not differ significantly from the observed data, *χ* ^2^(49) = 62.49, *P* < 0.093. Observed bead counts and attachment rates are therefore reported here (Tables 1 and 2). Model-parameters, fit-indices and selection-criteria for the negative binomial model are reported, together with significance values for each effect, in Table 3. The model’s intercept represents an arbitrarily chosen baseline for comparison, in this case the bike/long/moist condition. The log-coefficient for the intercept represents the estimated number of seeds in that condition once exponentiated, so exp(0.81) = 2.25 seeds in the bike/long/moist condition. As previously mentioned, model-estimated and actual number of seeds (2.9) did not significantly differ and, therefore, actual seed numbers are reported in Table 1. Condition effects in the model are calculated by adding relevant coefficients for main- and interaction-effects to the baseline before exponentiation. For example, to calculate the estimated number of seeds in the boot/long/wet condition, we added estimates for the Intercept, Vector, Soil Condition, and Vector × Soil Condition: exp(0.81 + 1.70 + 2.83 + (−2.23)) = 22.42 seeds, actual seed number = 24.7. Note that significant main effects should not be interpreted in the negative binomial model in the presence of significant interactions as they may be misleading. Condition analyses showed that, whilst there were significant effects of each of the three experimental factors (Vector, Soil Condition and Traversal Distance), all but one (Soil Condition × Traversal Distance) of the interactions between these factors were also statistically significant (Table 3). Owing to the complexity of these results and to avoid extensive statistical copy, results are summarised in the following plain text. Consistently more beads attached over the long traversal distance than over the short traversal distance; however the ratio of short to long was variable. More beads attached under wet conditions than under moist conditions, although again the ratio of wet to moist was variable. Generally, more beads attached to boots than to bike tires under the same conditions, but again the ratio was variable and this pattern reversed under the long wet conditions (Table 1). In summary, bead attachment was higher for longer traversals and under wet soil conditions. Bead attachment was generally higher on boots than on tires, except when traversal distance was long and the soil condition was wet. Mean % attachment rate of beads from total available (11,180) was very low over all treatment combinations, ranging from 0.07 % (SE = 0.02 %)–0.22 % (SE = 0.03 %) for boots and 0.00 % (SE = 0.00 %)–0.31 % (SE = 0.04 %) for tires (Table 1).

**Table 3:** Negative binomial model showing results of the three-factor analysis

	Log-coefficient (SE)	*z*	*P*
Intercept	0.81 (0.32)**	2.893	.004
Vector	1.70 (0.32)***	5.240	<.001
Soil condition	2.83 (0.33)***	8.944	<.001
Traversal distance	−1.59 (0.38)***	−4.125	<.001
Vector × Traversal distance	0.99 (0.36)**	2.994	.003
Vector × Soil condition	−2.23 (0.34)***	−5.994	<.001
Soil condition × Traversal distance	0.57 (0.31)	1.649	.099
*α* (dispersion parameter)	0.20		
Log-likelihood (LL)	−165.56, df = 8		
Akaike information criterion (AIC)	347.11, df = 8		
Bayesian information criterion (BIC)	363.32, df = 8		
Residual deviance	62.49, df = 49		

Reported are parameter estimates (log-coefficients and associated, robust standard errors), fit- and model selection indices (*LL*, *AIC*, *BIC*) and associated degrees of freedom (*df*)

** = significant at *P* < .01, *** = significant at *P* < .001

## Discussion

Our finding that bike tires had a lower propensity than boot soles to pick up beads under all conditions tested except over 150 m distance travelled in wet soil was initially surprising and counter-intuitive, given the tires’ larger overall surface area than the boot soles. However, the result that the bike tires tended to pick up fewer beads than boot soles makes sense, as the tread depth of the tires was shallower (2 mm) than that of the boots (sole 5 mm; heel 10 mm) and hence the beads/soil may not have adhered as tightly to the tires as they did to the bottom of the boot. This is supported by the observation reported during testing that beads attaching to boot soles were predominantly in the heel treads (see Results above). It may be that for shorter distances and/or dryer soils the potentially deeper and narrower tread of the boot soles meant that more beads were retained on boots, but that on a longer ride on wet soil, the greater surface area of the ti re becomes more important, allowing soil to attach over a greater area resulting in more beads attaching. Increasing the density of beads in the soil in a repeat experiment so there are fewer zeros and low numbers attaching may assist in testing this hypothesis.

It must also be remembered that beads were only picked up along with soil in our experiment. It is possible that in other circumstances, for example seeds growing on trackside vegetation and possessing traits affecting attachment on walkers’/riders’ clothing, for example differing morphology, mass and infructescence height might affect attachment rate, as might walkers’ and riders’ relative speed of travel along such tracks.

Our study gives the first published quantification of the propensity for attachment of a seed analog on bike tires, both in absolute terms and comparative to boot soles. It provides a comparison with the very small number of controlled experiments quantifying seed attachment rate on footwear, either directly or in a soil matrix, for a measured sampling effort (e.g., compare Mount and Pickering 2009; Wichmann et al. 2009). However, comparison of our results with previous studies must be considered relative to the respective studies’ very differing sampling protocols and to several important caveats which we detail below.

Our “long” test distance (≈150 m) was broadly comparable to that employed by Mount and Pickering (2009; Experiment 3) who experimentally tested seed attachment on a single pair of boots worn by a single walker over 100 m (*n* = 20). Their mean seed attachment quantity on boot uppers (excluding laces) and soles combined (number attaching specifically to soles unreported) was 60.5 (SE = 26.2) (trousered leg) and 71.4 (SE = 23.6) (bare leg). Our mean observed attachment quantity and variability were substantially lower, both for boots (7.7 [SE = 1.82]–24.7 [SE = 3.25]) and tires (0.00 [SE = 0.00]–34.6 [SE = 4.42]) under both moist and wet soil conditions (Table 1). However, these results are not directly comparable owing to very different sampling protocols employed: in the Mount and Pickering (2009) study (i) their walker traversed Australian alpine roadside vegetation, not a walking track; (ii) they measured direct seed attachment on the boots from plants and/or loose seed on the soil surface, not in the soil matrix; (iii) soil was “relatively dry” (moisture level not reported) and no soil was collected on the boots and; (iv) seed quantity available for attachment was unknown.

A key issue in all studies attempting to quantify seed attachment rates is ‘what constitutes a realistic soil seed density in natural areas?’ As previously noted, our experiment employed beads of comparable size, shape and density as the seeds used by Wichmann et al. (2009). Our “short” walking distance of 25 steps was also broadly comparable to their sampling protocol of 20 steps. However, as their study was primarily focused on seed dispersal distance, their sampling protocol design was designed to maximise seed attachment and their 20 steps were repeated ‘on the spot’ in each of two small [0.4 × 0.25 m^2^] trays containing (i) wetted soil (moisture % level not reported) and (ii) 100 seeds. They recorded high attachment rates, ranging from (Experiment 1: two seed species, one walker and boot type) 4–93 % attachment, mean 52% and 42 %, variability unreported and (Experiment 2: one seed species, 10 walkers, mix of walking/Wellington boots) 26–52 % attachment, mean % and variability unreported]. The authors noted that their sampling protocol did not match the “real situation” and that their recorded attachment rates were ‘probably artificially high’ (Wichmann et al. 2009, p. 525, 530). In comparison, our observed attachment rates on boots in the short distance test, under arguably more realistic “real world” conditions, were typically two orders of magnitude lower, with means ranging 0.07 % (SE = 0.02 %) –0.18 % (SE = 0.03 %). Attachment rates on bike tires over the same distance were lower still, with means ranging 0.00 % (SE = 0.00 %) –0.14 % (SE = 0.05 %) (Table 1).

## Caveats and Conclusion

Our study suggests potential benefits of a new methodology by which researchers might cost-effectively manipulate and test the effects of different influencing factors on initial seed attachment and transport rate in a soil matrix on boot soles and bike tires, both in absolute and comparable quantities. However, our results are subject to the following important caveats.

Firstly, we were using plastic beads as an analog for seeds, not real seeds. However, seeds of different species exist in a wide range of morphologies and adhesive qualities, masses and sizes and we therefore argue that our beads can be considered as a representative analog of real seeds on all three parameters except for the small hole centring the beads. The only two previous controlled studies of direct seed attachment on boots that we found (e.g. Falinski 1972; Mount and Pickering 2009) recorded such diversity, although neither was able to quantify attachment rate in proportion to a known available seed quantity, unlike our study. Only one other controlled study (Wichmann et al. 2009) has tested attachment propensity in a soil matrix on boot soles for pre-selected, specified seed types (2: *Brassica oleracea* ssp. and *Brassica nigra*): as previously noted our beads were specifically selected to be a comparable size and shape to seeds used in that study.

Secondly, although our use of the circular test track allowed us to simulate a realistic walking and riding pattern and beads were available for attachment from on top of/within shallow surface soil, similar to conditions likely to be the case in a natural environment, the methodology employed in the “long” (≈150 m) test distance, necessitating repeatedly walking/riding the same track, meant that some beads might have become attached, detached and subsequently reattached on boot soles and bike tires. Although we were unable to quantify this, we regularly observed soil dropping back onto the track from both boots and bike tires during circuits. This was especially marked for the bike under “wet” conditions, with soil (possibly containing beads) picked up on the tires often unable to pass through the caliper brake pads and subsequently ejected back onto the track. This issue was probably less likely to occur for boot soles because, as previously noted, boots predominantly tended to pick up soil and beads in the heel treads, with soil tightly compacted and requiring beads to be physically extracted by the researchers, with very few beads (estimated <5 %) attaching to the remainder of the soles. In defence of the sampling methodology, however, we argue:

(i) This study is a pilot test of a potentially very flexible and cost-effective sampling methodology; the possible occurrence and scale of the potential attach/detach/reattach issue would benefit from further testing.
(ii) The % of beads attaching from the available bead reservoir on a ‘short’/single circuit was very low overall (0.07–0.18 % boot soles; 0.00–0.14 % bike tires); this suggests that the probability of the same individual beads re-attaching during multiple circuits is likely to be very low.
(iii) The ‘short’/single circuit distance test is unaffected by this potential issue and estimates of seed attachment over longer distances can therefore be arrived at via simple multiplication.

Thirdly, time and funding limits meant that our small-scale experiment used the same, single walker/rider for all tests and only one pair of boots and one bike. Boots and bike tires obviously come in a very wide variety of materials, sizes and tread patterns and these may affect seed attachment rate. Different walking/riding behaviour of individuals may also have an effect. Wichmann et al. (2009) found seed attachment rate differed significantly among different walkers and shoe type (walking boots vs. Wellington boots), although not among different shoe sizes.

For the above reasons, our results presented here are necessarily case-specific and cannot be generalised more widely to define the absolute relative propensity for seed attachment and transport rate in a soil matrix on boot soles and bike tires. We nevertheless suggest that the methodology as trialled here shows significant promise for researchers to use it more comprehensively to test the attachment rate of different seed types under a range of densities and soil conditions across a variety of different compounds and sizes of boot soles and bike tires, in a way that is cost-effective and that reflects real-world walker and biker behaviour.
